# Multimodal magnetic resonance imaging in the diagnosis of cervical cancer and its correlation with the differentiation process of cervical cancer

**DOI:** 10.1186/s12880-023-01104-4

**Published:** 2023-09-29

**Authors:** Heng Meng, Xin Guo, Duo Zhang

**Affiliations:** Department of Radiology, Affiliated Hospital of Beihua University, Jilin, 132011 China

**Keywords:** Cervical cancer, Magnetic resonance imaging, Diffusion weighted imaging, Intravoxel incoherent motion, Diffusion kurtosis imaging

## Abstract

**Purpose:**

This study seeks to evaluate the value of MRI (Magnetic resonance imaging) diffusion weighted images (DWI), diffusion kurtosis imaging (DKI) and intravoxel incoherent motion (IVIM) in the diagnosis of cervical carcinoma.

**Methods:**

Seventy-nine cases of cervical cancer (CC group) (39 cases of squamous carcinoma (SCC group) and 40 cases of adenocarcinoma (ACC group)) and 30 cases of healthy controls (HC group) were included in this study. All the subjects were informed of the purpose of this study. The study was approved by the Ethics Committee of Beihua University Hospital, Jinlin, China. In this study, images were acquired based on a 3T MR scanner (Ingenia; Philips, Best, the Netherlands) and measured the imaging parameters by DWI, IVIM and DKI techniques. The parameters were obtained by Philips post-processing workstation, DKE and IVIM. These ROIs (region of interest) were manually drawn on each parameter mapping image by MRI physicians. Finally, SPSS 23.0 statistical software was used for data analysis.

**Results:**

The ADC (apparent diffusion coefficient) value of M group was lower than that of N group, and the difference was statistically significant (*P* < 0.05). The D (true diffusion coefficient) value, D*(pseudo diffusion coefficient) value, f (perfusion fraction) value, MD (mean diffusivity) value, and ADC value in the SCC group were lower than those in the ACC group with statistically significant differences (*P* < 0.05). The MK (mean kurtosis) value was higher than that of the ACC group, and the difference was statistically significant (*P* < 0.05). Compared with the HC group, the ADC values, D values, MD values of group CC group were lower, and the D* values, f values, MK values were higher; all the parameters were statistically significant (*P* < 0.05). The higher the differentiation degree of cervical cancer, the higher ADC values, D values, MD values, and the smaller D* values, f values, MK values. The difference of ADC values, D values and MK values was statistically significant (*P* < 0.05). MK value had the best diagnostic efficiency in the differential diagnosis of cervical cancer with low and medium differentiation, high and low differentiation (*P* < 0.05). There was no significant difference in the f value between high and low differentiation cervical cancer (*P* > 0.05). There was no significant difference in the MD value between low and high differentiation cervical cancer (*P* > 0.05). The strongest correlation between MK values (*r* = 0.796) and the degree of pathological differentiation of cervical cancer is positively correlated. The D values, MD values, and ADC values are negatively correlated with the degree of pathological differentiation of cervical cancer.

**Conclusion:**

The ADC value of DWI parameters has important diagnostic value for different menstrual states of cervical cancer. The parameter values of DWI, IVIM, and DKI can be used to differentiate cervical cancer from normal cervical tissue, and thus have important diagnostic value for differentiating pathological types of cervical cancer. This means that these parameter values may have great significance in the differential diagnosis of cervical cancer with different degrees of pathological differentiation. The pathological differentiation degree of cervical cancer is significantly positively correlated with the MK value in the parameter values of DWI, IVIM, and DKI, while negatively correlated with the D value, MD value, and ADC value.

## Introduction

Cervical cancer is a higher incidence of malignant tumor in women all over the world. Therefore, it is very important to improve the early detection rate of cervical cancer, preoperative staging and pathological classification for the treatment and prognosis of cervical cancer. IVIM (voxel incoherent motion) theory divides the microscopic movement of organism into two types. One type is slow motion, described by D, it represents the movement of intercellular fluid and the rotation produced by extravascular (non blood), which is the diffusion of action. The other is rapid movement, the random distribution of blood in blood circulation network is described from macro perspective by D* description. The f and D* are perfusion parameters, which can reflect the degree of tumor vascular richness. DKI (diffusion peak imaging) is based on non Gaussian distribution model to quantitatively analyze the degree of inhomogeneity and the diffusion limit of real water molecules. The purpose of this study is to evaluate the complexity of the microstructure of biological tissue. DKI sequence and IVIM can quantitatively reflect the characteristics of lesions from diffusion and perfusion. Therefore, they can provide more comprehensive information for the diagnosis and prognosis of the disease. This study focuses on the differential diagnosis of cervical cancer and the degree of pathological differentiation of DWI, IVIM and DKI sequence.

## Patients and methods

### Patients

Seventy-nine cases of cervical cancer (CC group) and 30 cases of healthy controls (HC group) were collected. Cervical cancer patients were divided into two groups: One consists of 39 cases of cervical squamous carcinoma (SSC group). It included 10 cases of high differentiation, 18 cases of moderate differentiation and 11 cases of low differentiation. The other consists of 40 cases of cervical adenocarcinoma (ACC group). It included 9 cases of high differentiation, 19 cases of moderate differentiation and 12 cases of low differentiation.

According to the presence or absence of menopause, 68 patients were divided into menopause group (M group); 11cases of non menopausal period (N group).

The inclusion criteria of cervical cancer cases were as follows: (1) the patients were primary cervical cancer; (2) the patients had never received drug or radiation therapy before MRI examination; (3) the age was between 30 and 80.

The inclusion criteria of normal female volunteers were as follows: (1) the volunteers were identified as having a normal cervix by ultrasound or magnetic resonance imaging ; (2) before MRI examination, the volunteers had never received drugs, radiation and other treatments; (3) the age was between 30 and 80.

### Methods

All patients underwent T1WI(T1 weighted image), T2WI(T2 weighted image), DWI (diffusion weighted images), DKI (diffusion kurtosis imaging) and IVIM (intravoxel incoherent motion) sequence scanning on 3.0T MRI machine (Ingenia; Philips, best, the Netherlands), and the scan sequence parameters were as follows (Table 1).

**Table 1:** MRI scanning parameters

Name	TR(ms)	TE(ms)	FOV(mm×mm)	b(sec/mm^2^)
T1WI	676	20	230 × 189	
T2WI	3772	90	224 × 224	
DWI	6000	72	220 × 220	0, 400, 800, 1200, 1600
DKI	6692	61	220 × 220	0, 500, 1000, 1500, 2000
IVIM	6000	54	220 × 220	0, 5, 10, 20, 40, 80, 160, 300, 600, 800, 1000, 2000

The parameters were obtained by Philips post-processing workstation, DKE and IVIM. These ROIs were manually drawn on each parameter mapping image by MRI physicians. The range of interest was 50–80 mm^2^. Workspace post-processing software was used to measure DWI parameters ADC values, IVIM parameters (D, D*, f) and DKI parameters (MD, MK) were used as the final values of lesions. All the subjects of interest were selected with the help of two radiologists with more than 10 years’ experience.

### Statistical analysis

All the measured data were analyzed by SPSS 23.0. The measurement data is expressed as $\overline{\mathrm x}$± s. T-test was used to verify the average level of each parameter in cervical cancer group and normal cervical group. ROC curve was used to determine the optimal threshold value, specificity and sensitivity of parameter identification. Univariate analysis of variance was used to compare the differences of IVIM and DKI parameters between cervical cancer with different degrees of differentiation. LSD-t test was used to compare the ability of each parameter to distinguish the degree of cervical cancer differentiation. The difference was considered statistically significant when *P*-value was lower than 0.05.

## Results

1. Comparison of DWI, IVIM, and DKI parameters among different menstrual states of cervical cancer cases

The ADC value of M group was lower than that of N group, and the difference was statistically significant (*P* < 0.05). There was no statistically significant difference in D value, D* value, f value, MD value, and MK value between M group and N group (*P* > 0.05) (Table 2).

**Table 2:** Comparison of DWI, IVIM, and DKI parameters between M group and N group

	D (×10^−3^mm^2^/s)	D* (×10^−3^mm^2^/s)	f (%)	MD (×10^−3^mm^2^/s)	MK (×10^−3^mm^2^/s)	ADC (×10^−3^mm^2^/s)	
M group	0.801 ± 0.086	12.462 ± 1.139	0.202 ± 0.031	0.812 ± 0.061	0.909 ± 0.171	0.812 ± 0.061	
N group	0.876 ± 0.168	12.152 ± 2.508	0.213 ± 0.727	1.012 ± 0.091	0.902 ± 0.291	1.012 ± 0.091	
t	-0.787	0.262	-0.357	0.422	0.034	-6.011	
*P*	0.469	0.805	0.738	0.692	0.974	< 0.001

2. Comparison of DWI, IVIM, and DKI parameters in cervical squamous carcinoma (SCC group) and adenocarcinoma (ACC group)

The D value, D* value, f value, MD value, and ADC value in the SCC group were lower than those in the ACC group, with statistically significant differences (*P* < 0.05). The MK value was higher than that of the ACC group, and the difference was statistically significant (*P* < 0.05) (Table 3).

**Table 3:** Comparison of DWI, IVIM, and DKI parameters in SCC group and ACC group

	D (×10^−3^mm^2^/s)	D* (×10^−3^mm^2^/s)	f (%)	MD (×10^−3^mm^2^/s)	MK (×10^−3^mm^2^/s)	ADC (×10^−3^mm^2^/s)	
SCC group	0.760 ± 0.081	11.572 ± 1.129	0.190 ± 0.021	1.161 ± 0.240	0.982 ± 0.192	0.810 ± 0.062	
ACC group	0.931 ± 0.072	13.876 ± 0.847	0.242 ± 0.047	1.510 ± 0.361	0.762 ± 0.109	0.960 ± 0.115	
t	-4.539	-4.737	-4.282	-2.642	3.288	-4.279	
* P*	< 0.001	0.001	< 0.001	0.017	0.004	< 0.001

3. Comparison of DWI, IVIM and DKI parameters between healthy controls (HC group) and cervical cancer (CC group)

**Fig. 1:**
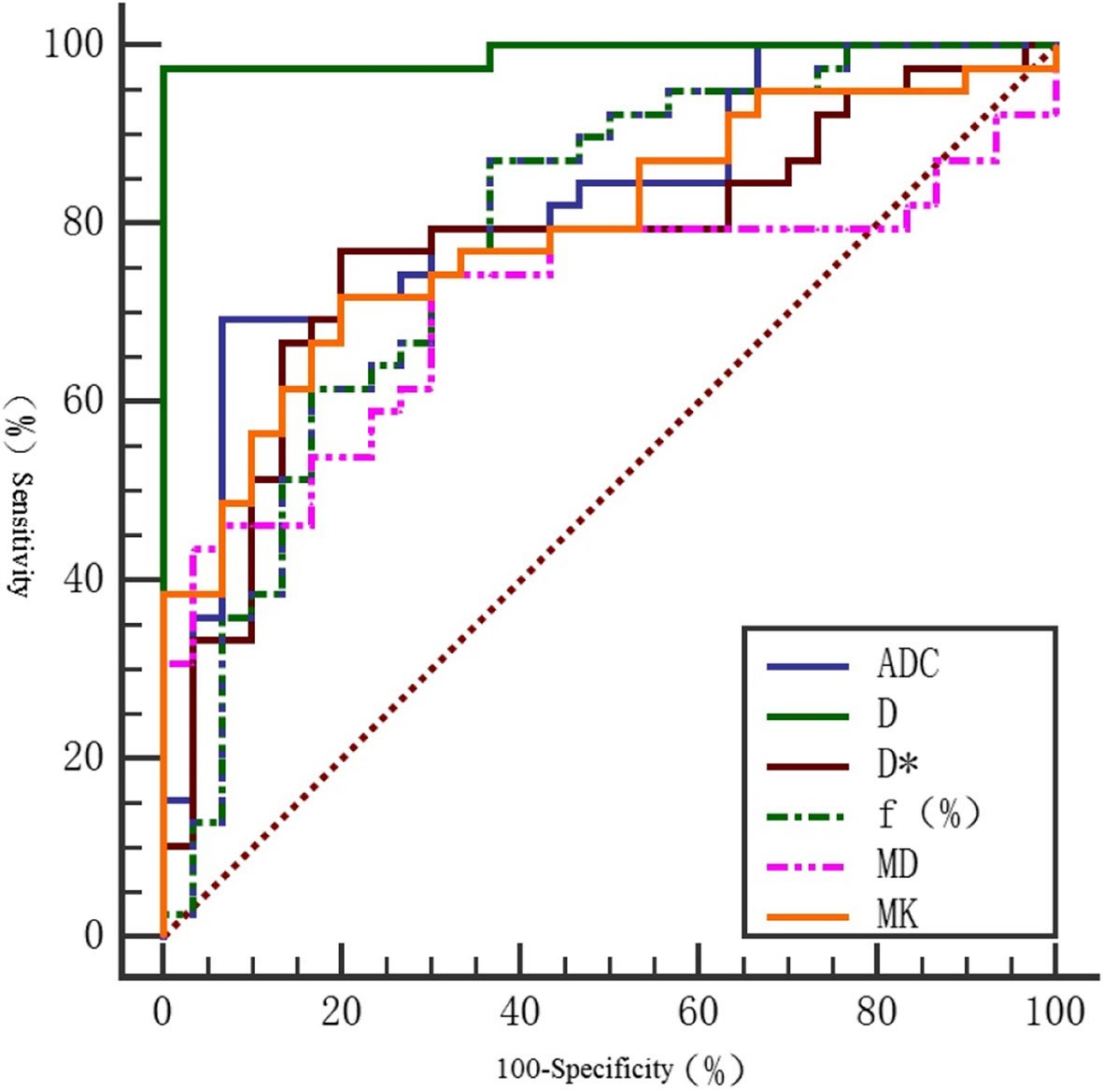
ROC curve of DWI, IVIM and DKI in cervical cancer and normal cervical tissue

**Fig. 2:**
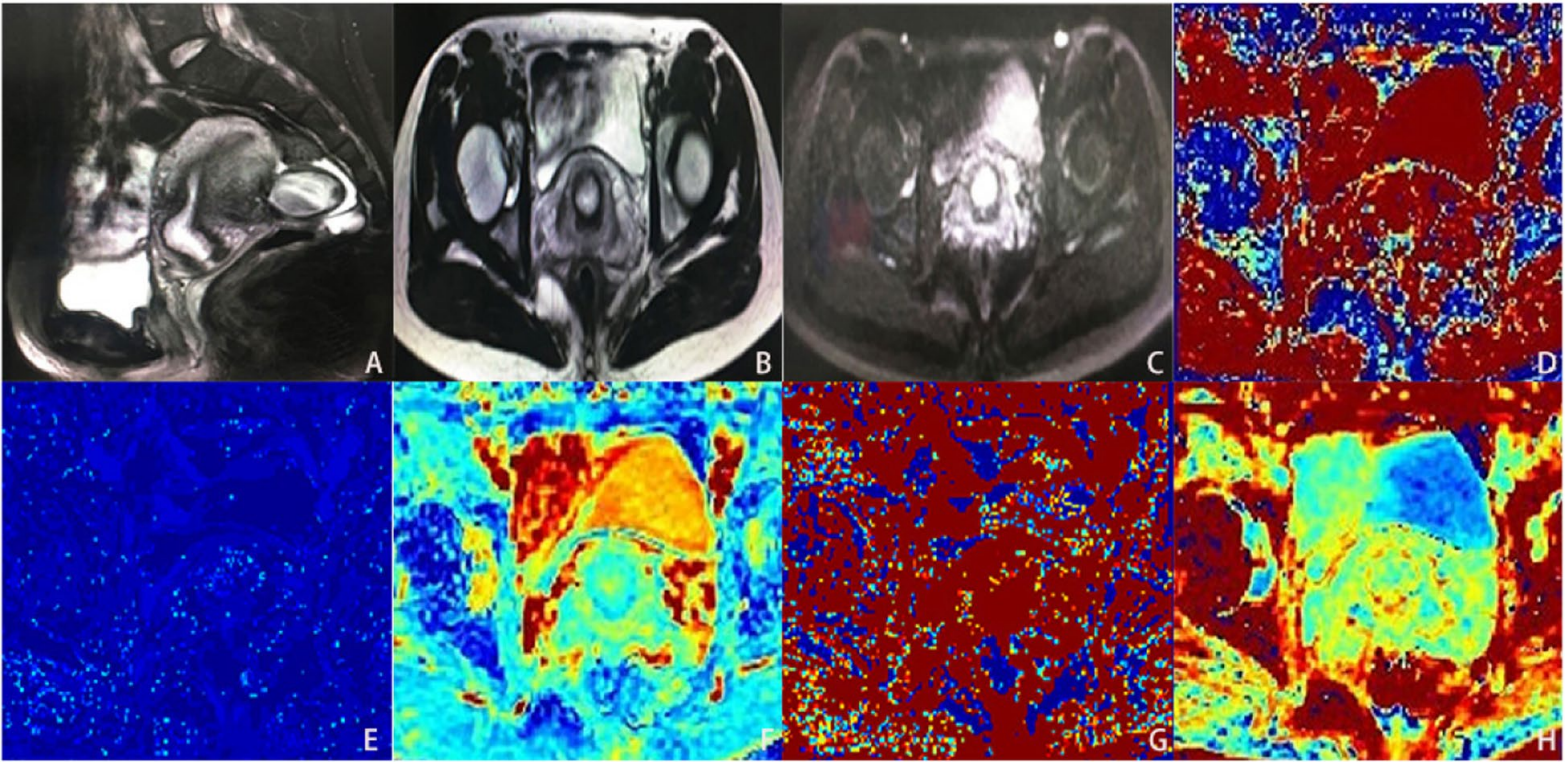
Normal cervical tissu. **A** is sagittal T2WI. **B** is axial T2WI. **C** is DWI (b = 800s / mm2). **D** is the pseudo color image of parameter D in IVIM. **E** is the pseudo color image of parameter D* in IVIM. **F** is the pseudo color image of parameter MD in DKI. **G** is the pseudo color image of parameter f in IVM. **H** is the pseudo color image of parameter MK in DKI

The ADC, D and MD values of CC group were lower than those of HC group. The area under ROC curve (AUC) of D value was the largest (0.991). The sensitivity is 100%. The specificity is 97.4%. The diagnostic threshold is 0.903 × 10^−3^ mm^2^/ s. The diagnostic efficiency of MK values is second largest. The area under the ROC curve (AUC) of MK is 0.870. The sensitivity is 90.9%. The specificity was 82.1% The diagnostic threshold was 1.025 (Tables 4 and 5) (Figs. 1 and 2).

**Table 4:** Comparison of DWI, IVIM and DKI parameters between HC group and CC group

	D (×10^−3^mm^2^/s)	D* (×10^−3^mm^2^/s)	f (%)	MD (×10^−3^mm^2^/s)	MK (×10^−3^mm^2^/s)	ADC (×10^−3^mm^2^/s)	
HC group	1.281 ± 0.162	10.338 ± 1.078	14.137 ± 5.252	1.703 ± 0.353	0.818 ± 0.110	1.029 ± 0.128	
CC group	0.731 ± 0.136	11.260 ± 1.041	19.012 ± 4.207	1.353 ± 0.608	1.009 ± 0.234	0.880 ± 0.115	
t	15.330	3.314	-4.282	2.814	4.118	5.052	
* P*	< 0.001	0.001	< 0.001	0.006	< 0.001	< 0.001	

**Table 5:** Comparison of ROC curves of DWI, IVIM and DKI

Parameter	Sensitivity (%)	Specificity (%)	Yoden index	Threshold	AUC (95%CI)	*P*
D (×10^−3^mm^2^/s)	100.0	97.4	0.974	0.903	0.991 (0.971, 1.000)	< 0.001
D* (×10^−3^mm^2^/s)	76.9	80.0	0.569	10.883	0.773 (0.658, 0.887)	< 0.001
f (%)	87.2	63.3	0.505	15.115	0.785 (0.672, 0.891)	< 0.001
MD (×10^−3^mm^2^/s)	70.0	74.4	0.444	1.629	0.711 (0.587, 0.829)	0.003
MK (×10^−3^mm^2^/s)	90.9	82.1	0.731	1.025	0.870 (0.759, 0.982)	< 0.001
ADC (×10^−3^mm^2^/s)	93.3	69.2	0.625	0.8962	0.821 (0.722, 0.9210)	< 0.001

4. Comparison of DWI, IVIM and DKI parameters of cervical cancer with various differentiation

The higher the differentiation degree of cervical cancer, the higher ADC, D, MD values, and the smaller D*, f, MK values. There were statistically significant differences in ADC, D and MK values between two groups of cervical cancer with different degrees of differentiation (*P* < 0.05). However, there was no significant difference in D*, f value between moderately and highly differentiated cervical cancer (*P* > 0.05). There was no significant difference in MD value between poorly differentiated and moderately differentiated cervical cancer (Table 6).

**Fig. 3:**
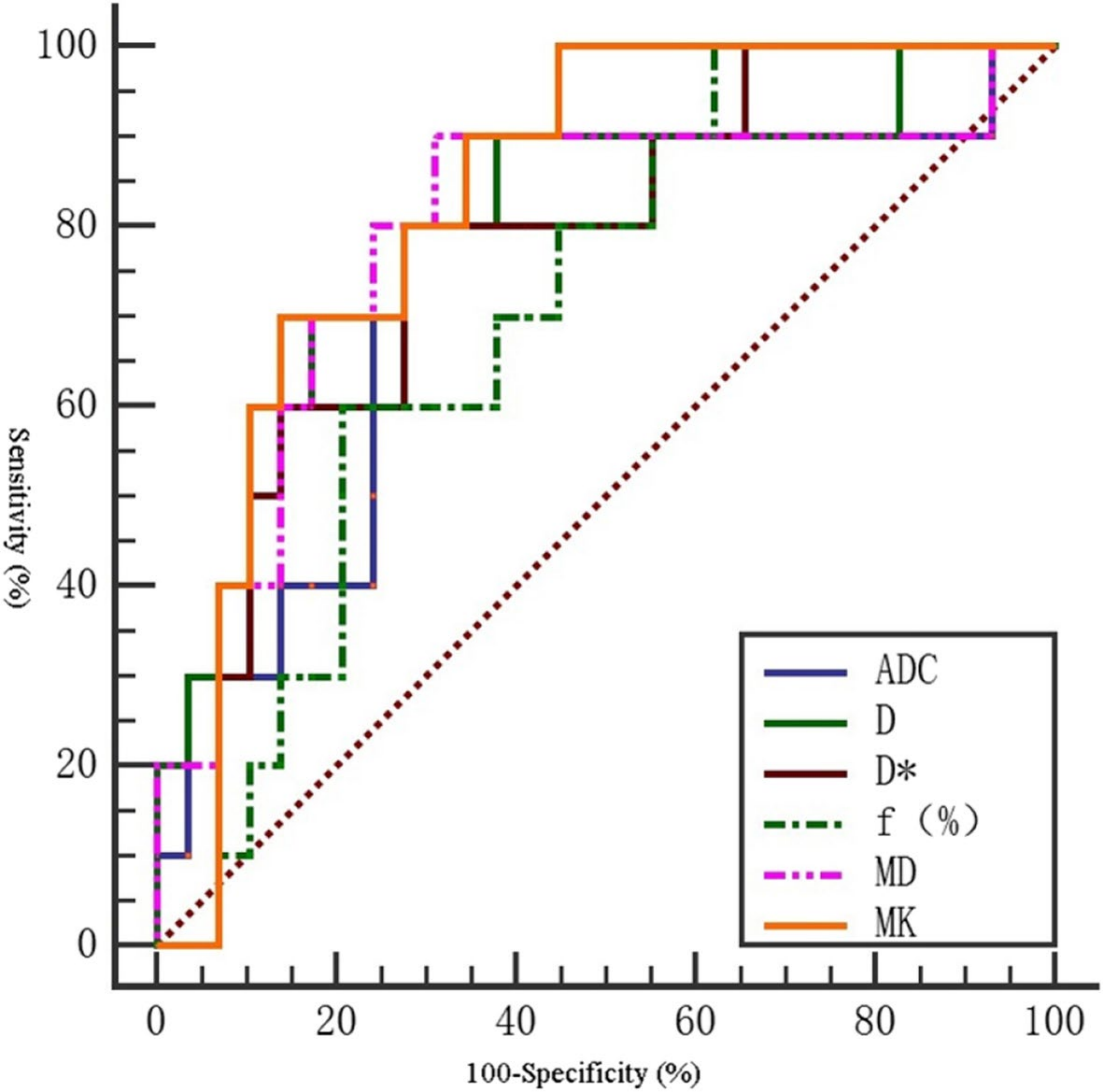
The ROC curve of DWI, IVIM and DKI in differentiating high and low differentiation cervical cancer

**Fig. 4:**
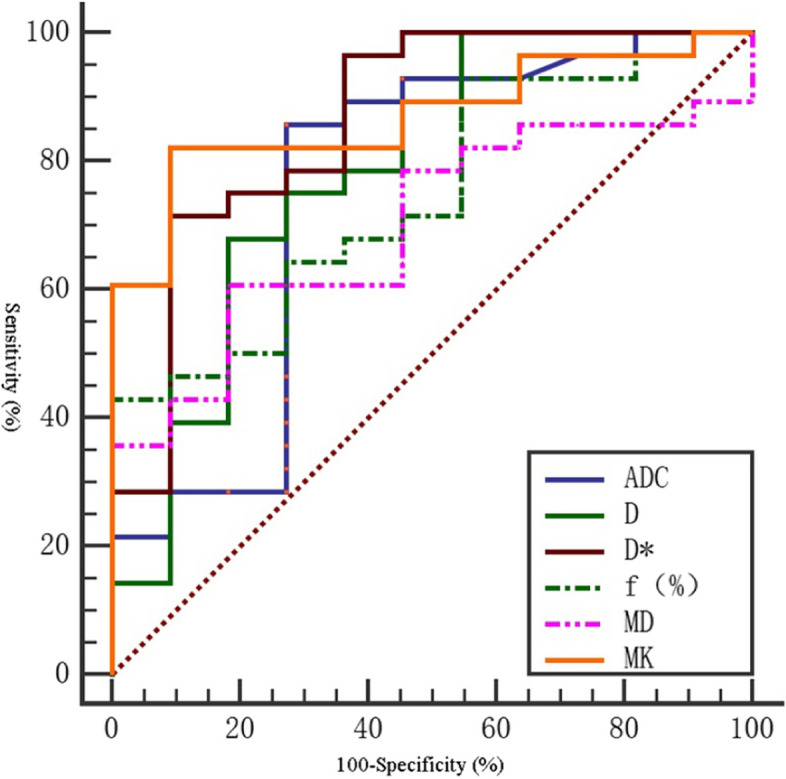
The ROC curve of DWI, IVIM and DKI in differentiating low and moderately high differentiation cervical cancer

**Fig. 5:**
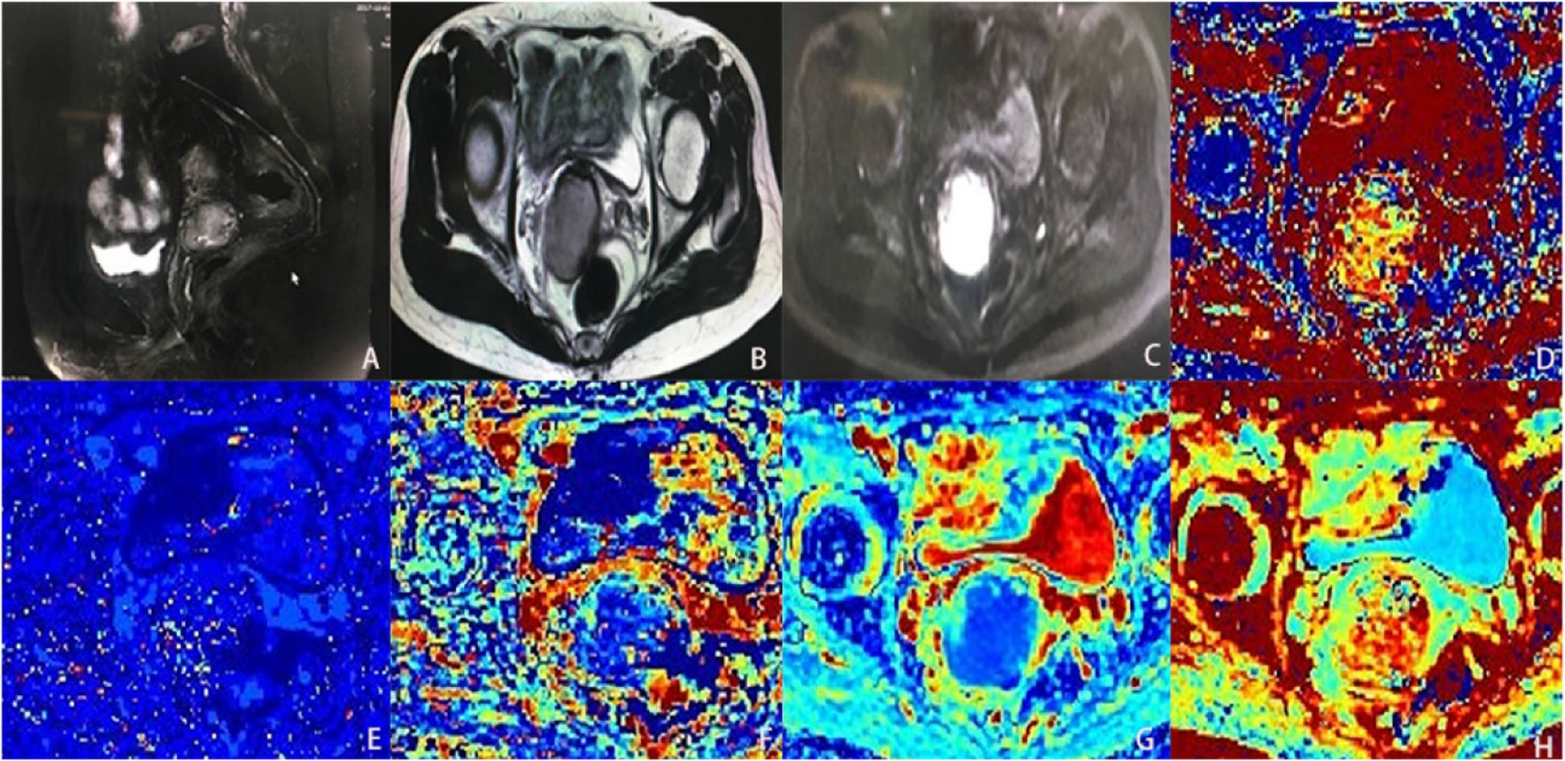
Moderately differentiated squamous cell carcinoma of the cervix. **A** is sagittal T2WI, and the tumor tissue showed slightly high signal intensity. **B** is axial T2WI, and the tumor tissue showed slightly high signal intensity. **C** is DWI (b = 800s / mm2), and the tumor tissue showed slightly high signal intensity. **D** is the pseudo color image of parameter D in IVIM, and the cancer tissue is yellowish. **E** is the pseudo color image of parameter D* in IVIM, and the cancer tissue is of partial color. **F** is the pseudo color image of parameter f in IVM, and the cancer tissue is bluish. **G** is the pseudo color image of parameter MD in DKI, and the cancer tissue is bluish, and the cancer tissue is bluish. **H** is the pseudo color image of parameter MK in DKI, and the cancer tissue is reddish

In the differential diagnosis of high and moderately low differentiation cervical cancer, the area under the ROC curve (AUC) of MK value was the largest (0.831). The sensitivity was 86.2%. The specificity was 70%. The diagnostic threshold was 0.897. The area under the ROC curve (AUC) of D value was (0.800). The sensitivity was 70.0%. The specificity was 82.8%. The diagnostic threshold was 0.771 × 10^−3^mm^2^ /S (Table 7). In the differential diagnosis of low and moderately high differentiation cervical cancer, the area under the ROC curve (AUC) of MK value was the largest (0.870). The sensitivity was 90.9%. The specificity was 82.1%. The diagnostic threshold was 1.025. The area under the ROC curve (AUC) of D* value was 0.864. The sensitivity was 90.9%. The specificity was 71.4%. and the diagnostic threshold was 11.223 × 10^−3^mm^2^/S (Table 8). There was no significant difference in f value between high and moderately low differentiation cervical cancer (*P* > 0.05). However, there was no significant difference in MD value between low and moderately high differentiation cervical cancer (*P* > 0.05) (Tables 7 and 8) ( Figs. 3, 4 and 5).

**Table 6:** Comparison of DWI, IVIM and DKI parameters in high, medium and low differentiated cervical cancer

	D (×10^−3^mm^2^/s)	D* (×10^−3^mm^2^/s)	f(%)	MD (×10^−3^mm^2^/s)	MK (×10^−3^mm^2^/s)	ADC (×10^−3^mm^2^/s)
Low(L)	0.636 ± 0.127	12.163 ± 0.908	21.623 ± 3.825	1.068 ± 0.331	1.213 ± 0.288	0.805 ± 0.098
Medium(M)	0.731 ± 0.07	11.015 ± 0.967	18.430 ± 4.600	1.278 ± 0.612	1.970 ± 0.208	0.890 ± 0.101
High(H)	0.834 ± 0.167	10.705 ± 0.657	17.185 ± 2.41	1.801 ± 0.628	0.855 ± 0.080	0.984 ± 0.120
F	7.412	8.429	3.691	4.898	9.530	4.760
P	0.002	0.001	0.035	0.013	< 0.001	0.015
L vs. M	0.043	0.002	0.041	0.329	0.002	0.042
L vs. H	< 0.001	0.001	0.024	0.005	< 0.001	0.004
M vs. H	0.032	0.379	0.428	0.022	0.0141	0.197

**Table 7:** The ROC curve of various parameters for differentiation of high and low differentiation cervical cancer

Parameter	Sensitivity (%)	Specificity (%)	Yoden index	Threshold	AUC (95%CI)	*P*
D (×10^−3^mm^2^/s)	70.0	82.8	0.528	0.771	0.800 (0.631, 0.969)	0.005
D* (×10^−3^mm^2^/s)	72.4	80.0	0.524	11.198	0.769 (0.606, 0.931)	0.012
f(%)	79.3	60.0	0.393	17.277	0.707 (0.540, 0.874)	0.054
MD (×10^−3^mm^2^/s)	80.0	69.0	0.490	1.373	0.793 (0.612, 0.974)	0.006
MK (×10^−3^mm^2^/s)	86.2	70.0	0.562	0.897	0.831 (0.700, 0.962)	0.002
ADC (×10^−3^mm^2^/s)	90.0	65.5	0.555	0.870	0.752 (0.567, 0.963)	0.019

**Table 8:** The ROC curve of various parameters for differentiation of low and moderately high differentiation cervical cancer

Parameter	Sensitivity (%)	Specificity (%)	Yoden index	Threshold	AUC (95%CI)		*P*
D (×10^−3^mm^2^/s)	75.0	72.7	0.477	0.698	0.786 (0.610,0.962)		0.006
D* (×10^−3^mm^2^/s)	90.9	71.4	0.623	11.223	0.864 (0.723,1.000)		< 0.001
f(%)	100.0	57.1	0.429	17.277	0.744 (0.582,0.905)		0.019
MD (×10^−3^mm^2^/s)	60.7	81.8	0.425	1.268	0.698 (0.532,0.864)		0.057
MK (×10^−3^mm^2^/s)	90.9	82.1	0.731	1.025	0.870 (0.759,0.982)		< 0.001
ADC (×10^−3^mm^2^/s)	85.7	72.7	0.584	0.810	0.755 (0.560,0.950)		0.014

5. Correlation analysis between DWI, DKI, IVIM parameters and pathological differentiation of cervical cancer

**Fig. 6:**
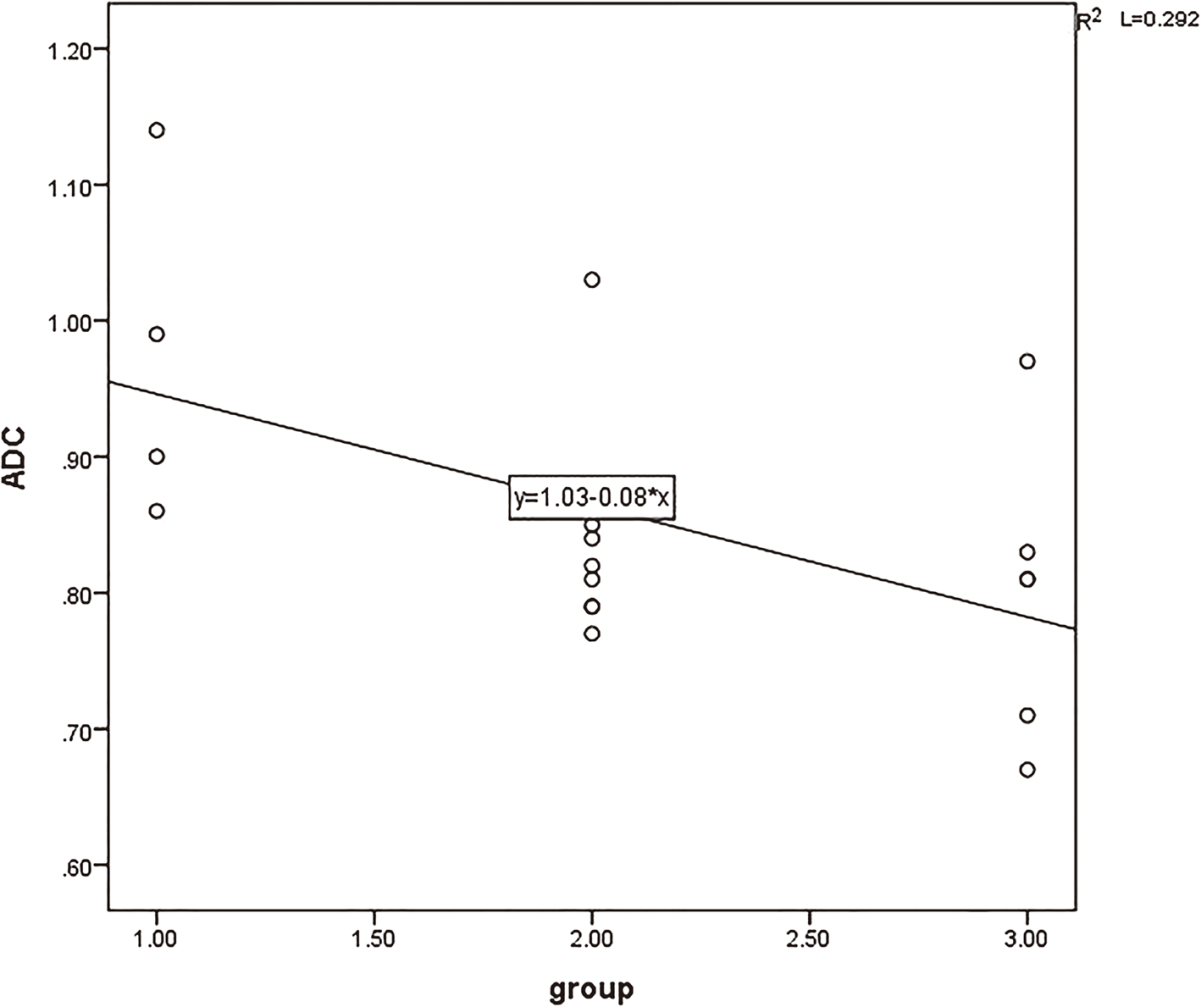
The correlation between MK value and pathological differentiation degree of cervical cancer (*r* = 0.796)

**Fig. 7:**
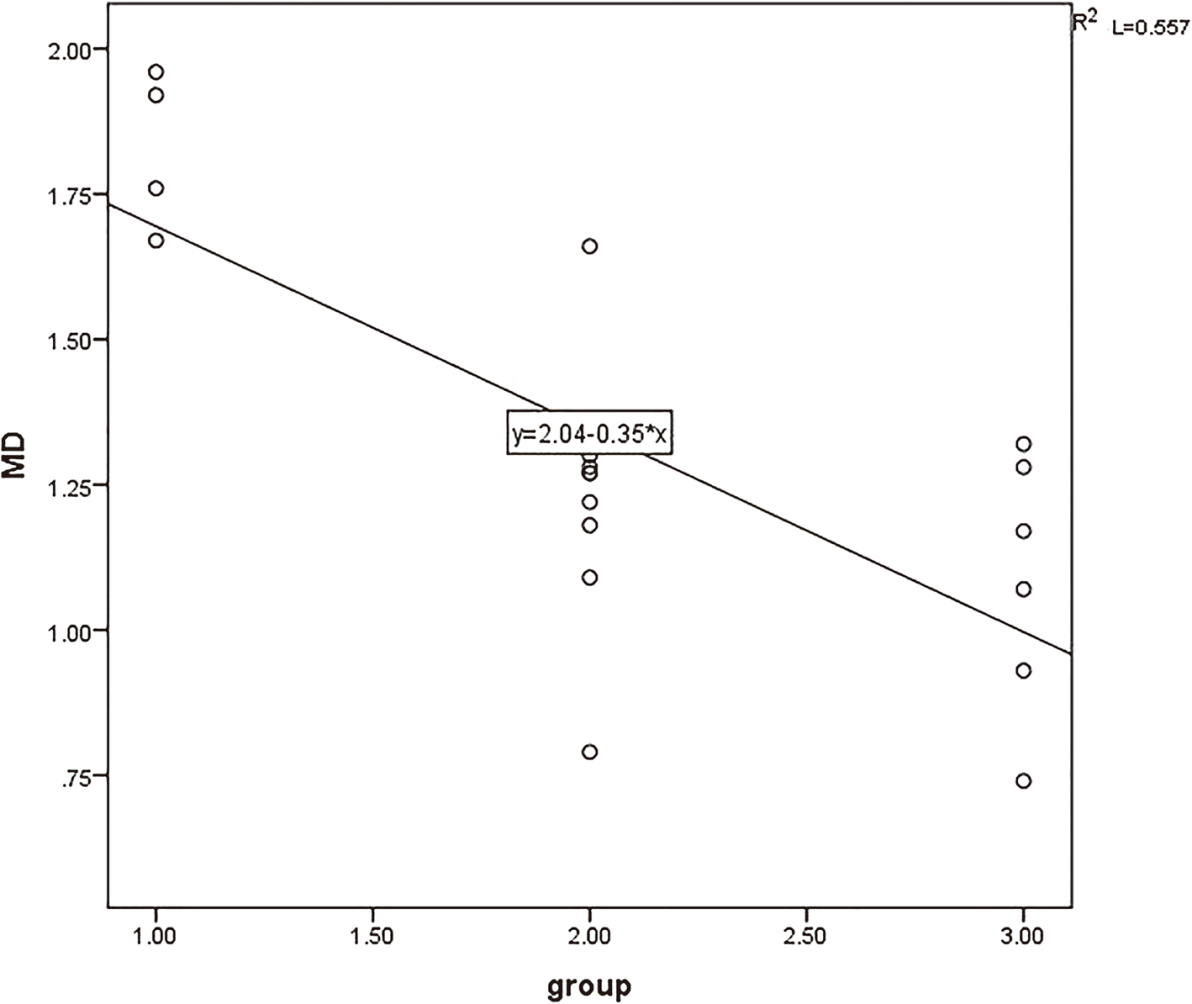
The correlation between ADCvalue and pathological differentiation degree of cervical cancer (*r *= -0.543)

**Fig. 8:**
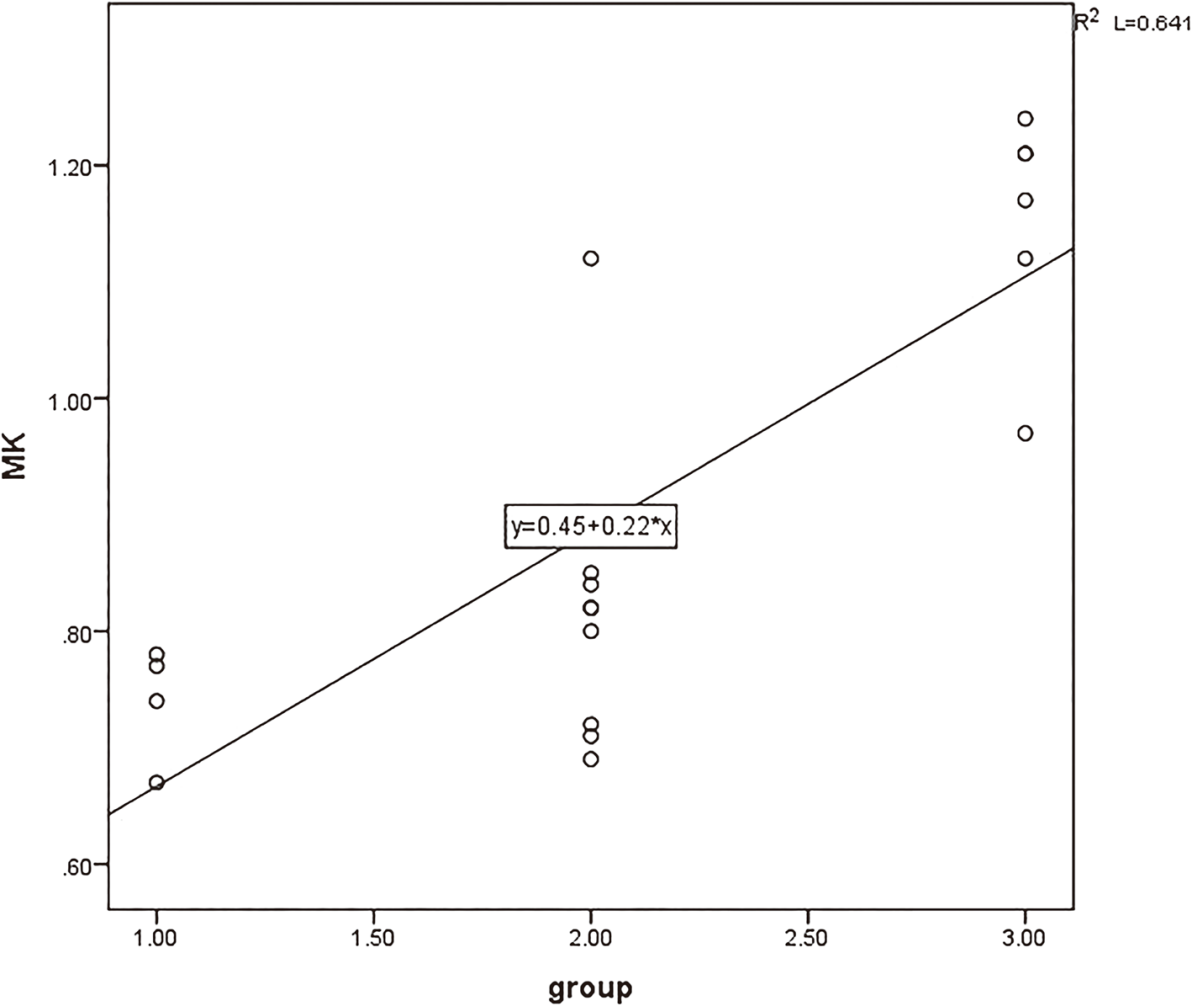
The correlation between D value and pathological differentiation degree of cervical cancer (*r* = -0.676)

**Fig. 9:**
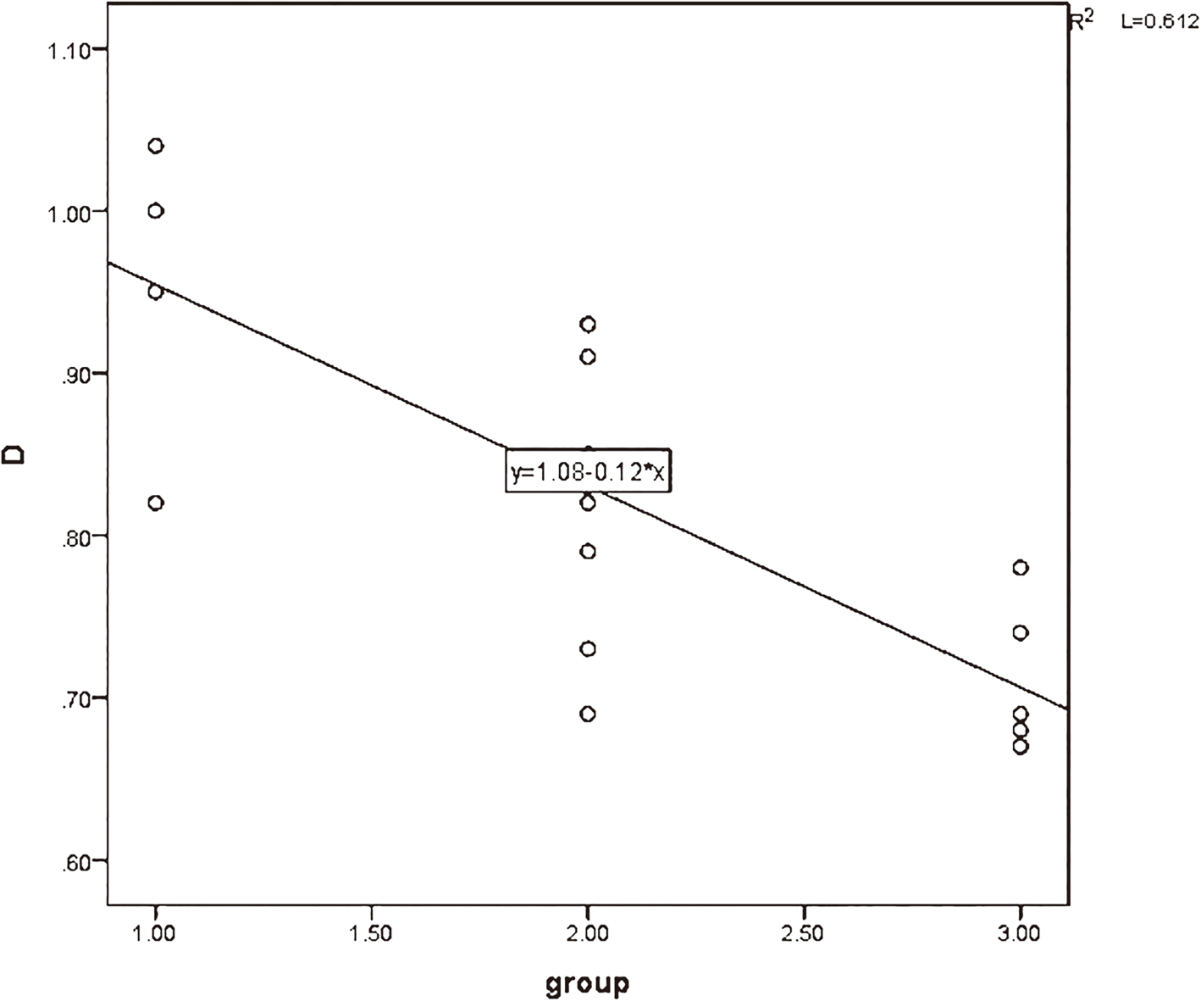
The correlation between MD value and pathological differentiation degree of cervical cancer (*r *= -0.785)

The strongest correlation between MK values (*r* = 0.796) and the degree of pathological differentiation of cervical cancer is positively correlated. The D values, MD values, and ADC values are negatively correlated with the degree of pathological differentiation of cervical cancer Figs. 6, 7, 8 and 9.

## Discussion

Cervical cancer is the second most common malignant tumor among women, and it still ranks the first in developing countries. It is important to correctly diagnose and determine the stage of cervical cancer.

The general basis of conventional DWI is the isotropy of water molecules [1]. The main principle of DWI is to analyze and judge the change of ADC value of tissue. ADC value is of great significance in evaluating the invasiveness of malignant lesions, tumor cell density, tumor pathology, differentiation degree and clinical treatment effect of tumor [2–4].

In voxel incoherent motion imaging (IVIM) refers to the translational motion that presents the distribution of velocity in direction or amplitude within a given voxel and within the measurement time. IVIM can be used as a method to evaluate tissue diffusion and perfusion in vivo [5]. At present, IVIM technology has three main parameters: D value, f value and D* value.

DKI technology is based on the complexity of the local tissue structure and mentality of the region of interest in vivo. Based on the DKI diffusion and kurtosis index of non Gaussian diffusion weighted model, it can distinguish the grade and stage of lesions [6]. DKI contains two parameters of MK value and MD value.

The study shows that there was significant difference in ADC values between the menopausal and non menopausal groups of cervical cancer, indicating that menstrual conditions have a significant impact on ADC values [7]. It may be the result of the hormone content and blood flow perfusion status [8].

There was no statistical difference in D value, D* value, and f value between different menstrual states groups. The reason is that different menstrual states did not affect the synthesis of intracellular macromolecules, so there was no significant impact on the D value.

Menstrual states should have had a certain impact on cell blood flow perfusion. However, in this experiment, the difference was not statistically significant. The reason may be that the sample size in this experiment was small, so different menstrual states did not significantly change the D* value and f value.

Previous studies have conducted qualitative studies on cervical cancer with various pathological features by measuring the ADC value of lesions, indicating that ADC values have important value to identify the morphology of cervical squamous cell carcinoma and adenocarcinoma [9].

The results of this study showed that the level of ADC in the adenocarcinoma group was significantly higher than that in the squamous cell carcinoma group, which was due to the rapid proliferation of squamous cell carcinoma tissue cells, fewer extracellular spaces, and significant limitations on the diffusion of extracellular water molecules. This resulted in a more significant decrease in ADC levels. As the density of squamous cell carcinoma cells is higher than that of adenocarcinoma, the diffusion limitation of water molecules is more significant. This resulted in lower MD values and higher MK values [10].

Adenocarcinoma tissue has a stronger ability to generate microvasculature and a higher density of capillaries, while squamous cell carcinoma tissue has a higher cell density, which reduces the microcirculation perfusion of squamous cell carcinoma tissue, thus reducing the D* and f values.

In the comparison of DWI, IVIM and DKI parameters between cervical cancer group and normal cervical group, the D value and ADC value of cervical cancer group were lower than those of normal cervical group. The number of cells per unit volume of cervical cancer tissue increased significantly, the diffusion of water molecules was hindered, and the ADC value decreased significantly, which could distinguish normal cervical tissue from cervical cancer tissue, and the number of cells in tissue increased significantly. The same is true for the IVIM parameter of D value. With the growth of tumor cell nucleus, the diffusion of water molecules in cells was also affected, and the D value finally decreased.

The values of f value and D* value in normal cervical group were lower than those in cervical cancer group, and the values of D* value and f value in IVIM parameters reflected the microcirculation perfusion information [11]. In the tumor tissue, the rapid increase of cells, the supply of nutrient vessels also increased rapidly, resulting in a significant increase in blood perfusion of tumor tissue. Hence, the measured D* value and f value will also be significantly greater than the normal cervical tissue.

The MD value of cervical cancer group is lower than that of normal cervical group, which is related to the content of MD value. The MD value represents the correlation coefficient of water molecular diffusion [12]. It is an improved ADC value, which reflects the diffusion degree of water molecules. Its diagnostic performance is similar to ADC value [6]. Therefore, the parameter value of cervical cancer group is less than that of normal cervical group. The MK value of cervical cancer group was higher than that of normal cervical group because of the heterogeneity and excessive proliferation of cervical cancer cells [13]. The more water molecules spread, the higher the MK value [14, 15]. Comparing the parameters of DKI, IVIM and DWI, it is found that IVIM parameter of the D value has the best diagnostic efficiency and the highest specificity in differentiating normal cervical cancer from cervical cancer.

In the comparison of DWI, IVIM and DKI parameters among high, medium and low differentiation groups of cervical cancer, we found that there were significant differences in the D value and MK value among low, medium and high differentiation groups of cervical cancer. ROC curve analysis showed that the area under the curve of the MK value was the largest when identifying high, low and middle groups. The area under the curve of the MK value was the largest when the low and middle high groups were identified. The second is the D* value. The results showed that there was a negative correlation between the differentiation degree of cervical cancer and cell atypia. With the increase of differentiation degree, cell atypia decreased. At the same time, the number of organelles and the ratio of core to core decreased, and the diffusion limitation of water molecules weakened [16]. The ADC, D and MD values mainly reflect the diffusion of water molecules. With the free diffusion of water molecules inhibited, the values become smaller; The f value and D* values mainly reflect the perfusion conditions. The tumor growth rate increases with the increase of the degree of malignant transformation of tumor [17]. The more nutrients the tumor tissue needs, the more tumor vessels supply nutrients, and the higher f value and D* value. MK has become an index to measure the complexity of organizational structure. The lower the cell heterogeneity, the more complex the components in the tissue, and the higher the MK value [18].

In the correlation analysis between the parameters of DWI, DKI, and IVIM and the degree of pathological differentiation, it was found that the MK value had the strongest correlation and was positively correlated with the degree of pathological differentiation of cervical cancer. The reason is that based on non Gaussian theory, the MK value comprehensively characterizes the distribution of water molecules in the lesion area, which best reflects the true movement of water molecules in the organism.

The main limitation of the present study is the relatively small number of patients included in the study affecting the statistical results. Although cervical cancer is among the most common types of cancers of women, this study only included a small sample size due to the large time comsuption of DWI, IVIM and DKI. If the collection time is extended and the sample size is expanded, the results of this study will be more objective. In addition, other studies have found that DTI has the higher accuracy in the diagnosis of parametrial invasion by cervical cancer, and the correct definition of T staging allows for better management of patients with cervical cancer [19]. In future studies, the sample size should be expanded and the DTI-related research should be included.

## Conclusion

1. The ADC value of DWI parameters has important diagnostic value for different menstrual states of cervical cancer.
2. The parameter values of DWI, IVIM, and DKI can be used to differentiate cervical cancer from normal cervical tissue, and thus have important diagnostic value for differentiating pathological types of cervical cancer. This means that these parameter values may have great significance in the differential diagnosis of cervical cancer with different degrees of pathological differentiation.
3. The pathological differentiation degree of cervical cancer is significantly positively correlated with the MK value in the parameter values of DWI, IVIM, and DKI, while negatively correlated with the D value, MD value, and ADC value.

## Data Availability

The datasets used and/or analysed during the current study are available from the corresponding author on reasonable request.
